# Simulation and Operational Optimization of RO Based Desalination for Coal-Fired Power Plants’ Wastewater

**DOI:** 10.3390/membranes12050478

**Published:** 2022-04-29

**Authors:** Lu He, Yudong Xia, Chuang Li, Aipeng Jiang, Yan Zhao, Fengling Xie

**Affiliations:** 1 School of Automation, Hangzhou Dianzi University, Hangzhou 310018, China; 202060285@hdu.edu.cn (L.H.); 42072@hdu.edu.cn (Y.X.); lichuang_1006@163.com (C.L.); 211060052@hdu.edu.cn (F.X.); 2 Chn Energy Lucency Enviro-Tech Co., Ltd., Beijing 100039, China; 12030975@chnenergy.com.cn

**Keywords:** Coal-fired power plant wastewater, membrane, simulation, optimization, mechanistic model

## Abstract

Focusing on the problems of opaqueness and high energy consumption in coal-fired power plant wastewater recycling processes, this paper studies the simulation and operational optimization of coal-fired power plant wastewater treatment by taking a coal-fired power plant system in Inner Mongolia as an example. Firstly, based on the solution–diffusion theory, pressure drop, and osmotic concentration polarization, a mechanistic model equation for coal-fired power plant wastewater treatment is developed. Secondly, the equation fitness and equation parameters are calibrated to obtain an accurate model. Thirdly, the system is simulated and analyzed so as to obtain the influence and change trajectories of different feed flowrates, temperatures, pressures, and concentrations on various aspects of the system’s performance, such as water recovery rate, salt rejection rate, and so on. Finally, in order to reduce the operating cost of the system, an optimization analysis is carried out, with the lowest specific energy consumption and average daily operating cost as optimization goals, and the performance changes of the system before and after optimization under three different working conditions are compared. The results show that adopting the given optimal strategy can significantly reduce the system’s operational cost. This research is helpful for the digitization and low-carbon operation of coal-fired power plant wastewater treatment systems.

## 1. Introduction

With the advancement of industrialization and urbanization, the discharge of wastewater has become increasingly uncontrolled, resulting in a series of environmental problems and a global water shortage. As water usage is a key operational aspect of most industrial processes, the generation of a large amount of industrial wastewater is inevitable [1,2]. With the significant increase in the world’s energy consumption, in order to meet the demand for increasing electricity consumption, coal-fired power plants have been intensively utilized [3]. Water is an essential and fundamental resource in thermal power plants with various uses: as a make-up for boiler feed water, in the cooling of bearings and equipment, and in various plant services. Nowdays, China has formed a coal-based energy production structure with oil, natural gas, electricity, and other energies complementing each other. Coal-based electricity accounts for about 90% of energy production [4]. Therefore, a large amount of water is used in coal-fired power plants in China. It has become very important to treat and reuse the wastewater from coal-fired power plants, especially in Central and Western China, due to water shortages.

Compared with seawater, wastewater from coal-fired power plants has a high pH and COD (chemical oxygen demand) and a complex water quality, which make treatment and reuse very difficult [5]. Coal-fired power plants’ wastewater is mainly divided into two categories: saline wastewater and organic wastewater [6]. Saline wastewater comes from recycling systems, such as circulating water systems and chemical water station drainage, mainly containing inorganic salts, such as Cl^−^, SO_4_^2−^, Na^+^, and Ca^2+^. Based on TDS, it can be classified into low-saline and high-salt water. The TDS of low-saline water is usually less than 10,000 mg/L and that of high-salt water is usually 10,000~20,000 mg/L. The organic wastewater produced by coal-fired power plants comes from gasification process wastewater, coal-water slurry, and black water of dry coal-pulverized entrained flow gasification, etc. It mainly contains phenol, naphthalene, anthracene, and thiophene. These are all refractory organics with poor biodegradability and are highly difficult to treat, requiring biochemical treatment. Saline water makes up a large share of wastewater in coal-fired power plants, and thus more efforts should be made to desalinate and reuse the wastewater.

So far, the desalination methods for saline wastewater treatment from coal-fired power plants mainly include membrane technology and thermal technology. Among them, membrane technology mainly includes microfiltration (MF), ultrafiltration (UF), nanofiltration (NF), and reverse osmosis (RO), etc. Thermal technology mainly includes multi-stage flash (MSF), multiple effect distillation (MED), and vapor compression (VC), etc. [7,8,9]. Reverse osmosis (RO) technology is extensively adopted in the treatment of wastewater from coal-fired power plants due to the advantages of high efficiency, simple operation, and maintenance of biological activity [10,11]. However, the operational efficiency of RO-based desalination systems is greatly affected by the feeding conditions of wastewater [12]. Therefore, it is of great significance to study the inherent operating characteristics of RO-based wastewater desalination systems so as to take effective measures to reduce the energy consumption and operational costs of these systems. For this reason, a mechanistic model is required to simulate and study the RO-based desalination process, at the same time enabling the operational optimization of the process.

Reverse osmosis seawater (SWRO) desalination is a typical resource treatment method of concentrated brine desalination. Malek et al. [13] established a model of a large-scale seawater desalination plant, including seawater intake and a pretreatment model, a high-pressure pump model, an energy recovery system model, and an RO process model. Based on these models, a replacement plan for membrane elements, system investment cost, and operating cost were provided, and an economic analysis was also carried out. Based on the solution–diffusion theory, Al-Bastaki et al. [14] established a mechanistic model of hollow fiber RO membranes, which considered the effects of pressure drops and concentration polarization. Considering the complex spatiotemporal variability of the membrane modules in the RO process, Karabelas et al. [15] proposed a “scale separation” global structured modeling method to develop a comprehensive SWRO system model. Al-Enezi et al. [16] gave the main structure of the RO system, which mainly includes one-stage, two-stage, and two-pass RO devices, and analyzed the effect of feed concentration and temperature on the salt rejection rate of the RO system. Oh et al. [17] gave a system model of SWRO based on solution–diffusion theory and studied the relationship between recovery rate and permeate flux and other performance aspects of the RO system. Kaghazchi et al. [18] presented an RO system mechanistic model of two desalination plants in the Persian Gulf region and analyzed the influence of operating parameters on system performance. Lu et al. [19] established a mathematical model of the desalination process unit, predicted the impact of operating parameters on the RO process under different conditions through simulation analysis, and gave the relevant economic model.

Using a detailed mathematical model of the RO-based desalination process, the optimized operation could be obtained by solving its corresponding optimization problem. In 2005, Marcovecchio et al. [20] established the mechanistic model of the hollow fiber membrane system and the economic model of the RO system, discretized the model by using the PR algorithm, and finally solved the optimization problem by GAMS. In 2007, Luo et al. [21] used the average value of the parameters of the membrane element model simplification of the differential solution operation. In 2013, Kim et al. [22] transformed the implicit mathematical model of the desalination process into an explicit model by converting the exponential function into a second-order polynomial and tested the accuracy, rapidity, and robustness of this method. In 2017, Gong et al. [23] used the finite element configuration method to discretize the differential equations in the membrane RO process model and optimized the solution by GAMS. In 2021, Blechschmidt et al. [24] used neural networks to solve the differential equations; this method is significant for RO system performance research.

In addition to using mechanistic modeling to analyze the RO process, some researchers have proposed a new method in recent years. In 2021, Cai et al. [25] used artificial neural network (ANN) and multilinear regression (MLR) techniques to model industrial RO concentrate processing. The performance of the models in predicting total organic carbon removal and sludge production in reverse osmosis concentrate treatment was evaluated and the ANN model’s predictive accuracy was validated. In 2021, Wang et al. [26] developed an ion transport model for RO, referred to as the solution–friction model, by rigorously considering the mechanisms of partitioning and the interactions among water, salt ions, and the membrane. Ion transport through the membrane is described by the extended Nernst–Planck equation, with the consideration of frictions between species. The model is validated using experimental measurements of salt rejection and permeate water flux in a lab-scale, cross-flow RO setup. Lastly, the pressure drop distribution across the membrane was analyzed by means of a framework. Bonny et al. [27] suggested a novel and efficient approach to determining transmembrane pressure using Deep Reinforcement Learning (DRL) and used a Deep Deterministic Policy Gradient (DDPG) agent to adjust the pressure across a membrane.

The above studies provide strong support for the modeling and solution of the RO process of membrane desalination, but most studies are mainly aimed at the desalination process of seawater or specific brines. It is known that there are few studies on modeling the wastewater treatment process of coal-fired power plants. Cai, Bonny, Salgado-Reyna, and Al-Obaidi et al. [25,27,28,29] used intelligent algorithms to model and analyze the RO process. These excellent studies used intelligent algorithms for research analysis; unfortunately, there may be some uncertainty in the analytic process. In this study, a mechanistic modeling method is applied to the treatment of coal-fired power plant wastewater so that the internal operation of the RO membrane can be seen intuitively. Most studies which establish mechanistic modeling are mainly aimed at the desalination process of seawater or specific brines, and there are few studies on the treatment of coal-fired power plant wastewater. Hence, a further improvement of the above models is required before they can be used for the simulation and operational optimization of the membrane treatment process of coal-fired power plant wastewater. Similarly, this study of coal-fired power plant wastewater treatment is relevant to the study of actual plants.

Therefore, in order to realize the efficient operation of coal-fired power plant wastewater treatment processes, this paper establishes a mechanistic model of an RO-based desalination system for coal-fired power plant wastewater treatment based on the theory of dissolution and diffusion, energy conservation, and material conservation. Then, aiming at the mismatch of model parameters, the parameters of the model are identified to obtain an accurate coal-fired power plant wastewater membrane treatment process model. Finally, the whole process is simulated and optimized to obtain the production process state and to determine mutual influence relationships, as well as the optimal operation strategy, so as to realize the optimal operation of the production process for the membrane treatment of coal-fired power plant wastewater.

## 2. Process Flow of Coal-Fired Power Plant Wastewater

**Figure 1:**
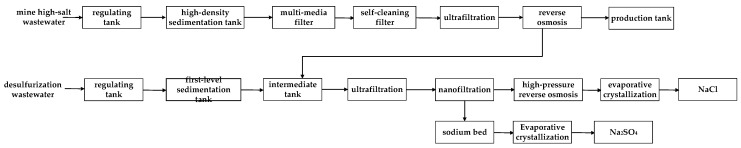
Typical process flow of coal-fired power plant wastewater.

RO modules are mainly divided into two types: spiral wound RO modules and hollow fiber RO modules. Compared with the hollow fiber RO module, the spiral wound RO module has a more compact structure and a larger effective membrane area per unit volume, so the spiral wound RO module is more popular [30]. Hence, this paper will analyze the spiral wound RO module. The process flow of a typical “zero-emission” coal-fired power plant wastewater treatment system is shown in Figure 1.

It can be seen from Figure 1 that the coal-fired power plant wastewater process mainly treats mine high-salt wastewater and desulfurization wastewater. The main process is as follows: the high-salt wastewater of the mine is extracted from the mine by the high-pressure pump and the impurities in the wastewater are removed by flocculation in the regulating tank; subsequently, the mine wastewater enters the high-density sedimentation tank. After dosing treatment, the wastewater passes through the multi-media filter, the self-cleaning filter, and ultrafiltration to remove other impurities. Then, it enters the RO modules to obtain fresh water and concentrated brine; after that the fresh water enters the RO production tank for further conditioning to become usable water and the concentrated brine enters the intermediate tank of the desulfurization wastewater process for further treatment. The desulfurization wastewater treatment process removes impurities through the adjustment tank and the first-level sedimentation tank and then the wastewater enters the intermediate tank. The intermediate tank mixes the concentrated brine produced by the mine’s high-salt wastewater process. The desulfurization wastewater is treated by ultrafiltration, nanofiltration, and high-pressure reverse osmosis. Finally, the desulfurization wastewater is evaporated and crystallized to obtain NaCl and NaSO_4_, realizing the “zero-emission” treatment of coal-fired power plant wastewater.

**Figure 2:**
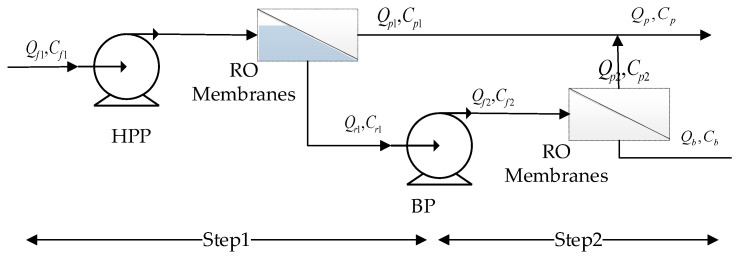
Flow chart of a two-stage RO plant.

With respect to the above process, this paper only analyzes the RO module in the mine high-brine treatment process. The arrangements of RO membranes in common RO modules mainly include one-stage, multi-stage, and multi-pass RO systems. This paper studies and analyzes the RO device with a two-stage system, and its flow chart is shown in Figure 2. After a series of treatments, the high-salt wastewater enters the RO device module and after the high-pressure pump pressurizes the high-salt water is pressed into the first-stage RO membrane for treatment to produce fresh water and concentrated bine water. The concentrated brine enters the second-stage RO membrane through the booster pump for treatment. In the same way, fresh water and concentrated brine can be obtained. The fresh water produced from the two stages enters the RO production tank and the concentrated brine enters the desulfurization wastewater treatment process and is treated again to realize the resource utilization of coal-fired power plant wastewater. The first-stage RO module consists of 12 pressure vessels; the second-stage RO module consists of 7 pressure vessels. Six membranes are connected in series in each pressure vessel.

## 3. Modeling of the RO Process of Coal-Fired Power Plant Wastewater Treatment

**Figure 3:**
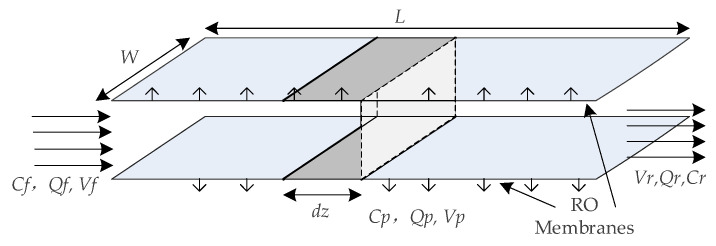
Schematic diagram of the inside of the feeding channel of the spiral wound RO modules.

Figure 3 shows the internal schematic diagram of the feed channel of spiral wound RO modules [12,18,31,32]. The feed water passes through the membrane channel to obtain fresh water and concentrated brine, respectively. The complete two-stage RO process mathematical model includes a process transport model and a system model [33,34].

### 3.1. RO Process Transport Model

Material conservation of solution:

(1) $$Q_{p1}=Q_{f}-Q_{r1}$$

Material conservation of solute:

(2) $$Q_{f}C_{f}=Q_{p1}C_{p1}+Q_{r1}C_{r1}$$

where *Q_f_* and *C_f_* are the flowrate and concentration of the feed water, respectively. *Q_p_*_1_ and *C_p_*_1_ are the flowrate and concentration of the water production side of the first-stage RO module, respectively. *Q_r_*_1_ and *C_r_*_1_ are the flowrate and concentration of the concentrated brine of the first-stage RO module, respectively.

The relationship between permeate flowrate and membrane parameters can be described using the following equation:

(3) $$Q_{p1}=n_{l}w\int_{0}^{L}J_{v1}dz=W\int_{0}^{L}J_{v1}dz$$

Solvent flux:

(4) $$J_{v1(z)}=A_{w}(\Delta P_{b1(z)}-\Delta \pi_{1(z)})$$

Solute flux:

(5) $$J_{s1(z)}=B_{s}(C_{m1(z)}-C_{p1(z)})$$

Transmembrane pressure:

(6) $$\Delta P_{b1(z)}=P_{b1(z)}-P_{p1(z)}$$

Osmotic pressure difference:

(7) $$\Delta \pi_{1(z)}=RT(C_{m1(z)}-C_{p1(z)})$$

Concentration polarization:

(8) $$\phi_{1(z)}=\frac{C_{m1(z)}-C_{p1(z)}}{C_{b1(z)}-C_{p1(z)}}=\exp(\frac{J_{v1(z)}}{k_{c1(z)}})$$

where *n_l_*, *w*, *L*, and *W* are the number of leaves, width, effective length of the RO module, and the total width of the RO module, respectively. *A_w_* and *B_s_* refer to the transport parameters of the RO membrane for solvent and solute, respectively. *P_b_*_1*(z)*_ and *P_p_*_1*(z)*_ are the brine pressure and produced water pressure at the *z* position of the first-stage RO module, respectively. *C_m_*_1*(z)*_ and *C_p_*_1*(z)*_ denote the brine concentration on the membrane surface at the *z* position of the first-stage RO module and the permeate water concentration on the permeate side, respectively. *R* and *T* are the gas constant and temperature, respectively. $\emptyset_{1\left(z\right)}\;$ is the concentration polarization phenomenon coefficient at the *z* position of the first-stage RO module. *C_b_*_1*(z)*_ is the brine concentration at the *z* position of the first-stage RO module. *k_c_*_1*(z)*_ is the mass transfer coefficient at the *z* position of the first-stage RO module.

The above equations are integrated as follows:

(9) $$J_{v1(z)}=A_{w}((\Delta P_{b1(z)}-P_{p1(z)})-RT(C_{m1(z)}-C_{p1(z)}))$$

(10) $$J_{s1(z)}=B_{s}\exp(\frac{J_{v1(z)}}{k_{c1(z)}})(C_{b1(z)}-C_{p1(z)})$$

(11) $$J_{s1(z)}=J_{v1(z)}C_{p1(z)}$$

The formula for calculating the mass transfer coefficient *k_c_*_1*(z)*_ of concentration polarization is as follows:

(12) $$Sh=\frac{k_{c1(z)}d_{e}}{D_{AB1(z)}}=k_{1}Re^{0.875}Sc^{0.25}$$

Reynolds coefficient:

(13) $$Re=\frac{\rho_{1\left(z\right)}V_{b1\left(z\right)}d_{e}}{\mu_{1\left(z\right)}}$$

Schmidt coefficient:

(14) $$Sc=\mu_{1(z)}/(\rho_{1(z)}D_{AB1(z)})$$

Here, *D_AB_*_1*(z)*_ is the dynamic viscosity. *Re* and *Sc* denote the Reynolds coefficient and the Schmidt coefficient, and *d_e_* is the equivalent diameter. *k*_1_ is the coefficient. $\rho_{1\left(z\right)}$ is the brine density at the *z* position of the first-stage RO module. *V_b_*_1*(z)*_ is the superficial feed velocity at the *z* position of the first-stage RO module. $\mu_{1\left(z\right)}$ is the dynamic viscosity of the solution at the *z* position of the first-stage RO module. The specific formulas of the above parameters are as follows:

Dynamic viscosity:

(15) $$D_{AB1(z)}=\gamma_{1}\cdot \exp(\gamma_{2}C_{b1(z)}-\frac{2513}{273.15+T})$$

The density of brine in the channel:

(16) $$\rho_{1(z)}=a_{1}M+\sqrt{a_{2}M^{2}+a_{3}MC_{b1(z)}}$$

The dynamic viscosity of brine in the channel:

(17) $$\mu_{1(z)}=\alpha \cdot \exp(\beta C_{b1(z)}-\frac{1965}{273.15+T})$$

Variable *M*:

(18) M=k−b⋅T

Here, $\gamma_{1}$ and $\gamma_{2}$ are the coefficients in the dynamic viscosity formula, which are related to the temperature and composition of the solution, etc. *a*_1_, *a*_2_, *a*_3_, α, and β are the coefficients in the regression equation of coal-fired power plant wastewater treatment, and the variable *M* is inversely proportional to the temperature *T*.

The relationship between the local parameters of the RO module is as follows:

Local solution mass conservation:

(19) $$Q_{b1(0)}=Q_{b1(z)}+Q_{p1(z)}$$

Local solute material conservation:

(20) $$Q_{b1(0)}C_{b1(0)}=Q_{b1(z)}C_{b1(z)}+Q_{p1(z)}C_{p1(z)}$$

where *Q_b_*_1(0)_, *Q_b_*_1*(z)*_, and *Q_p_*_1*(z)*_ refer to the feed water flowrate in the first-stage RO module, the brine water flowrate at the *z* position of the RO module, and the flowrate of the permeate water side, respectively. *C_b_*_1(0)_, *C_b_*_1*(z)*_, and *C_p_*_1*(z)*_ represent the first-stage feed water concentration, the brine concentration at the *z* position of RO module, and the permeate water side concentration, respectively.

Local flowrate:

(21) $$\frac{dQ_{b1(z)}}{dz}=-\frac{dQ_{p1(z)}}{dz}=-WJ_{v1(z)}$$

Pressure drop:

(22) $$P_{d1(z)}=\frac{1}{2}\rho_{1(z)}\int_{0}^{z}\lambda \frac{V_{b1(z)}^{2}}{d_{e}}dz$$

(23) $$\lambda =K_{\lambda }Re^{-0.3}$$

Local velocity:

(24) $$V_{b1(z)}=V_{b1(0)}-\frac{2}{\varepsilon_{sp}}\int_{0}^{L}\frac{J_{v1(z)}}{H}dz$$

Local salt concentration:

(25) $$C_{b1(z)}=\frac{V_{b1(0)}}{V_{b1(z)}}C_{b1(0)}-\frac{2C_{p1(z)}}{\varepsilon_{sp}V_{b1(z)}}\int_{0}^{L}\frac{J_{v1(z)}}{H}dz$$

Among them, *P_d_*_1*(z)*_ is the pressure drop at the *z* position of the first RO module, *k_λ_* represents the empirical value, *V_b_*_1(0)_ is the feed water flowrate in the first stage, $\varepsilon_{sp}$ is the membrane porosity, and *H* represents the height of the feed channel.

### 3.2. System Performance Model Equations

In the process of the RO modules shown in Figure 2, the RO modules of the two stages are of the same type, so the structure and principle of the membrane elements of the two stages are the same, and the above-mentioned transport model is also applicable to the second stage. The system model only needs to express the relationship between the feed water parameters of the second stage and the brine outlet of the first stage and the system performance relationship.

The parameters of the second stage of feed water flowrate are expressed as follows:

The second-stage feed water flowrate:

(26) $$Q_{f2}=\frac{Q_{r1}\cdot NP1}{NP2}$$

The second-stage feed water concentration:

(27) $$C_{f2}=C_{r1}$$

The second-stage feed water pressure:

(28) $$P_{f2}=P_{r1}+P\_boost$$

where *NP1* and *NP2* denote the number of pressure vessels in the first stage and the second stage of the RO system, respectively. *P_r_*_1_, *C_r_*_1_, and *Q_r_*_1_ are the pressure, concentration and flowrate of the first stage of brine treatment, respectively. *P_f_*_2_, *C_f_*_2_, and *Q_f_*_2_ refer to the pressure, concentration, and flowrate of the second-stage feed water, respectively. *P-boost* represents the pressure of the booster pump.

Combining the above equations, the key performance parameters of the RO system equations are as follows:

Water recovery rate:

(29) $$R_{ec}=\frac{Q_{p1}+Q_{p2}}{Q_{f}}\times 100\%$$

Salt rejection rate:

(30) $$R_{y}=(1-\frac{Q_{p1}\cdot C_{p1(L)}+Q_{p2}\cdot C_{p2(L)}}{Q_{f}\cdot C_{f}})\times 100\%$$

Specific energy consumption:

(31) $$SEC=\frac{P_{f}Q_{f}/(\varepsilon_{p}\cdot \varepsilon_{VFD})+P\_boost\cdot Q_{f2}/\varepsilon_{bp}}{Q_{p}}$$

Here, *Q_f_*, *C_f_*, and *P_f_* are the flowrate, concentration, and pressure of the feed water of the RO modules, respectively. *Q_p_*_1_ and *Q_p_*_2_ represent the outlet water flowrate of the first stage and the outlet water flowrate of the second stage, respectively. *C_p_*_1*(L)*_ and *C_p_*_2*(L)*_ are the final concentrations of the first- and second-stage waters, respectively. $\varepsilon_{p}$, $\varepsilon_{VFD},$ and $\varepsilon_{bp}$ refer to the mechanical efficiency of the high-pressure pump, the mechanical efficiency of the variable frequency drive, and the mechanical efficiency of the booster pump, respectively.

## 4. Model Calibration and Simulation Analysis

### 4.1. Model Parameter Calibration

Since the parameters of the seawater desalination model do not fit in the coal-fired power plant wastewater treatment model, the model is calibrated with the smallest variance between the model calculated data and the data output by the design software as the objective function. The optimization is solved by solvers, such as IPOPT and CONOPT of the GAMS platform [20]. Its specific optimization proposition is as follows:

Objective function:

$$\underset{A_{w},B_{s},K_{\lambda },k_{1},\gamma_{1},\gamma_{2}}{Min}E\left(\theta \right)=\underset{A_{w},B_{s},K_{\lambda },k_{1},\gamma_{1},\gamma_{2}}{Min}{\sum_{i=0}^{n}\omega_{i}\left(y_{icalc}-y_{imeas}\right)}^{2}$$

Such that:

$$\begin{array}{l} f(x,u,v)=0\\ A_{w}^{L}\leq A_{w}\leq A_{w}^{U}\\ B_{s}^{L}\leq B_{s}\leq B_{s}^{U}\\ K_{\lambda }^{L}\leq K_{\lambda }\leq K_{\lambda }^{U}\\ K_{1}^{L}\leq K_{1}\leq K_{1}^{U}\\ \gamma_{1}^{L}\leq \gamma_{1}\leq \gamma_{1}^{U}\\ \gamma_{2}^{L}\leq \gamma_{2}\leq \gamma_{2}^{U} \end{array}$$

where *y_icalc_* represents the calculated value of the model, *y_imeas_* represents the output value of the design software, $\omega_{i}$ represents the weight, the superscripts *L* and *U* refer to the lower and upper bounds of the parameter, and the f(x,u,v)=0 equation represents the RO process model of the system.

Through the optimization and solution of the above proposition, we can obtain the optimal model parameters and finally the correct mechanistic model for coal-fired power plant wastewater treatment.

In order to better study the modeling and optimization process of the system, this paper takes a coal-fired power plant wastewater resource utilization system in Inner Mongolia as an example. In the process flow chart shown in Figure 2, the RO device of this system is a two-stage membrane module. The RO modules of this system use the PROC10 from Hydranautics. The PROC10 membrane module is an enhanced, low-polluting, low-pressure RO composite membrane, which has the advantages of high salt rejection, a low pressure drop, less fouling, easy cleaning, and a long life. The parameters of the membrane module are shown in Table 1. The water quality parameters of the coal-fired power plant wastewater at the feed port of the RO system are shown in Table 2.

We established and calibrated the RO system model, as the model of Hydranautics’ design software IMSDesign is confidential, and the software is calculated off-line and cannot simulate the RO system in real time. When the plant was designed, it was simulated in advance through the IMSDesign software. Hence, the GAMS calculation data were first compared with the output data of the IMSDesign software, and the correctness of the model was verified based on this. The above data were used as the input of the GAMS platform and the IMSDesign software, respectively, and the results of the model calculation were outputted. In view of the different calculation methods of the model, only the final operating results of the RO system were compared, and the intermediate results of the RO system were no longer compared. The comparison results are shown in Table 3.

From the comparison of the model data before and after correction in Table 3, it can be seen that the calibrated parameters of the model improved the accuracy of the model. By comparing the calculated data of the calibrated model with the data of the design software IMSDesign, the maximum difference between the permeate concentration of each section and the final permeate concentration was 0.036 m^3^/h; the errors of the first and second stage were 15.7% and 1.2%, respectively; and the error of the feed water pressure was 2.2%. Only the first stage had a large error with respect to the concentration of the produced water; the rest of the errors were relatively small. In the actual operation of the power plant, these errors are acceptable, and the validity of the model is proved for the first time.

To further verify the correctness of the model, the actual data for the plant were compared with the output data of GAMS. Read the plant RO module feed water flowrate, the feed water pressure, and the feed pump outlet concentration as inputs to the GAMS simulation program. The feed concentration is converted by the read conductance value as shown in Equation (32).

(32) $$C_{f}=K*EC_{25℃}/1000$$

*K* represents the conversion coefficient, which is related to the impurity content in the solution, and the value here is 0.67. $EC_{25℃}$ represents the conductivity value converted to the reference temperature of 25 °C.

Three groups of available data are selected for calculation and comparison, among which the readable data of the power plant and the output data of GAMS are shown in Table 4. The feed water flowrate of the RO modules, the feed water pressure of the first stage of the RO modules, and the outlet concentration of the feed pump are used as the feeding conditions for GAMS simulation. From the table data, the errors of the RO module permeate water flowrate are 6.26%, 5.77%, and 1.32%, respectively. The errors of the feed water flowrate of the RO module booster pump are 7.59%, 2.41% and 16.43%, respectively. The errors of RO module concentrated water flowrate are 12.97%, 5.57%, and 2.50%, respectively. The permeate water pressure errors of the first stage of the RO modules are 0.05%, 4.53%, and 1.21%, respectively. The errors of the concentrated water pressure of the RO modules are 2.10%, 2.65%, and 2.10%, respectively. The reason for the errors between the power plant data and the GAMS output data may be the drift of the sensor, so the above comparison data once again clarify the correctness of the model.

### 4.2. Model Simulation Analysis

**Figure 4:**
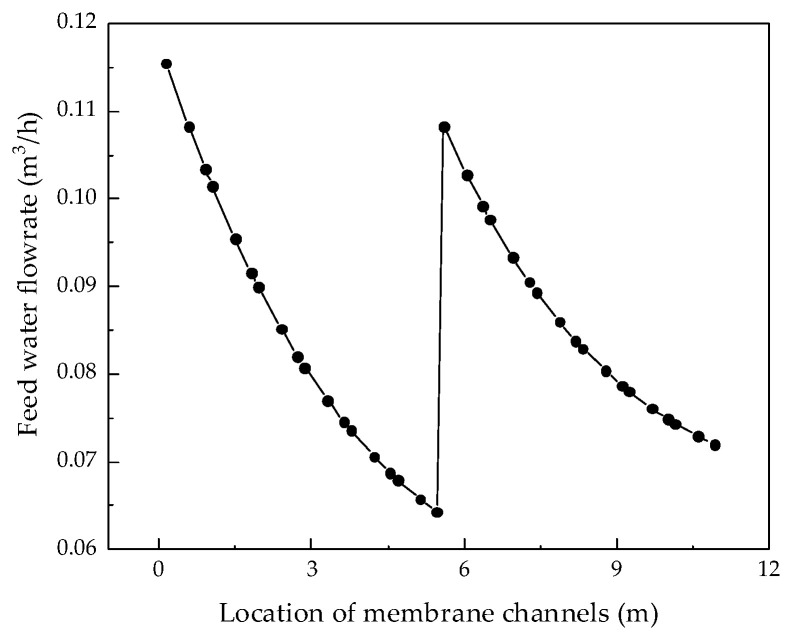
Variation curve of feed flow rate along the membrane channel.

**Figure 5:**
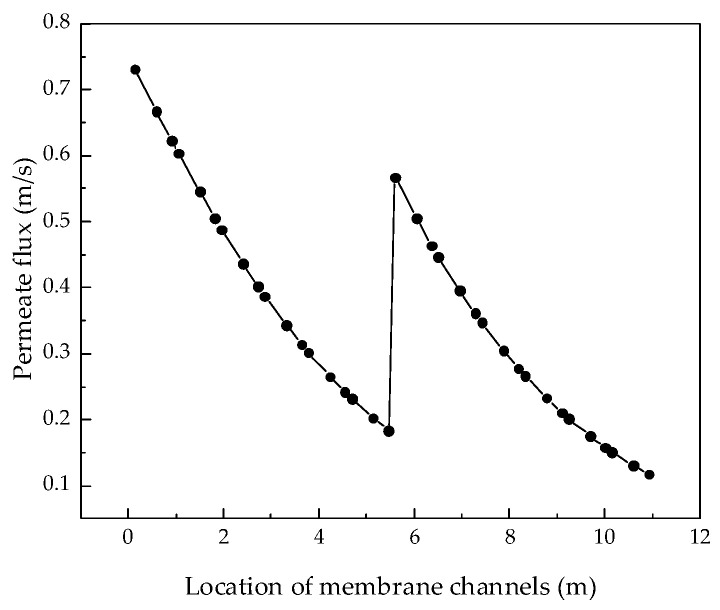
Variation curve of permeate flux along the membrane channel.

**Figure 6:**
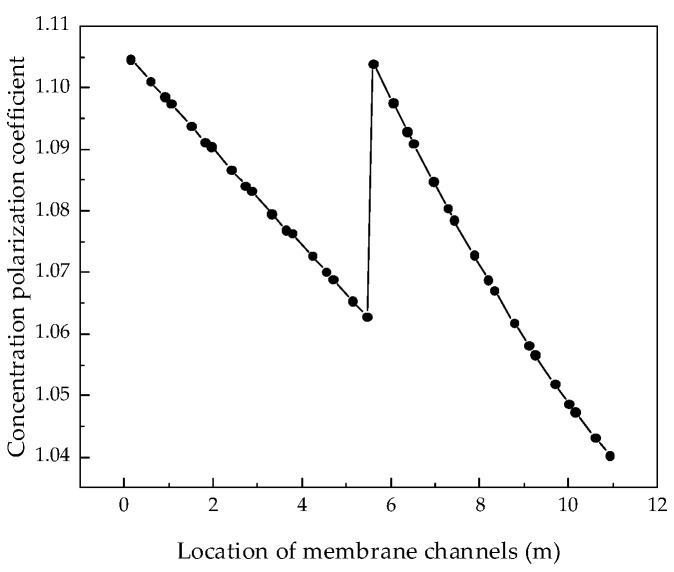
Variation curve of concentration polarization along the membrane channel.

**Figure 7:**
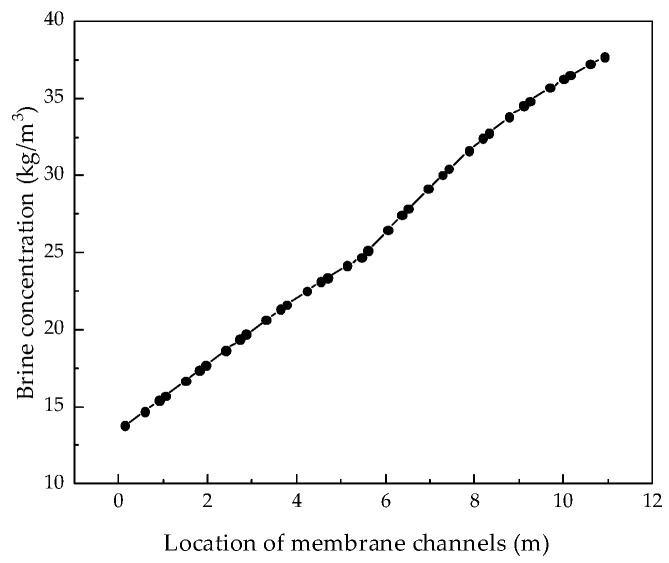
Variation curve of brine concentration along the membrane channel.

After verifying the validity of the model, the system performance changes corresponding to different parameters were simulated and calculated. Through the simulation, the internal states of the RO system can be displayed intuitively, so that those unmeasurable states can be displayed. Figure 4, Figure 5, Figure 6 and Figure 7 show the calculated results of the parameters given in Table 1 and Table 2 for the two-stage RO system. From the process flow chart shown in Figure 2, it can be noted that there is a booster pump between the first stage and the second stage. This booster pump of the RO system enables the feed flowrate, permeate flux, concentration polarization, and other pressure-related parameters to jump in the second stage. In order to fully demonstrate the performance changes of the RO system, all the following research results show the calculation data for the first and second stages are shown in same figure.

It can be seen from the simulation results that the feed flowrate, permeate flux, and concentration polarization of the two-stage membrane modules all show an approximate linear decreasing trend. The main reason is that when the feed water is treated into fresh water and concentrated brine, as the osmotic pressure of the RO modules increases, the driving force remains unchanged and the feed flowrate, permeate flux, and concentration polarization decrease. Since the brine concentration has little correlation with the increase in feed water pressure, Figure 7 shows that the brine concentration in the membrane channel continues to increase along the membrane channel.

The above analysis has obtained the internal operation of the system. In order to understand the characteristics of the system more comprehensively, the following analyzes the influence of the coal-fired power plant wastewater flowrate, temperature, pressure, and concentration at the inlet of the RO modules on the system performance. In the analytic process, other parameters are fixed and only the analyzed variable parameter is changed; then, the simulation calculation is carried out so as to observe the different effects of different parameters of the coal-fired power plant wastewater at the entrance of the RO modules on the system’s performance.

#### 4.2.1. Effect of Feed Water Flowrate on System Performance

**Figure 8:**
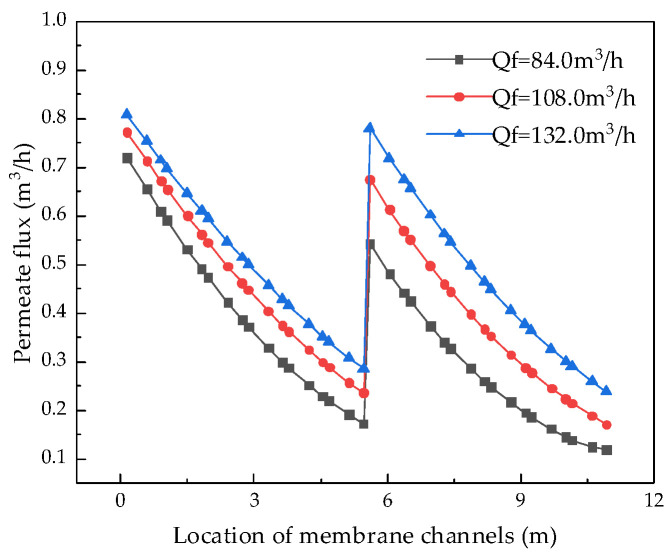
Effect of changes in feed water flowrate on permeate flux.

**Figure 9:**
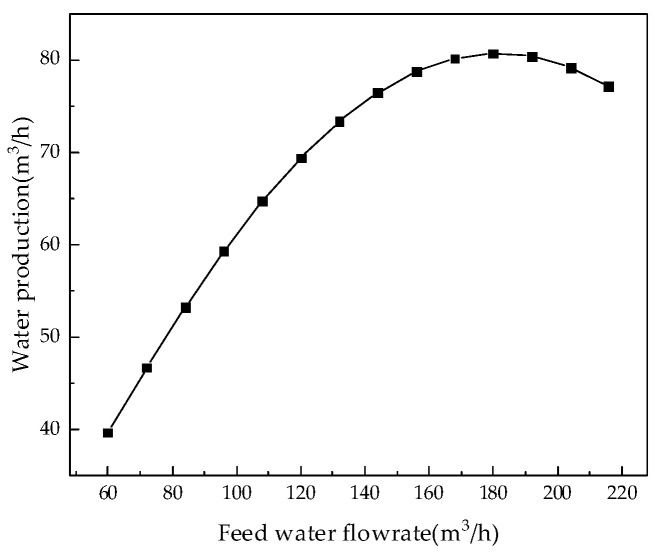
Effect of changes in feed water flowrate on water production.

**Figure 10:**
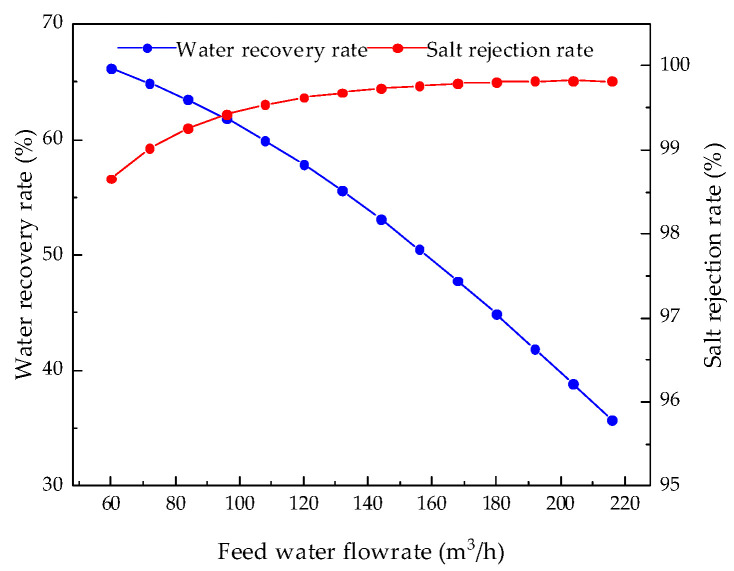
Effects of changes in feed water flowrate on water recovery rate and salt rejection rate.

**Figure 11:**
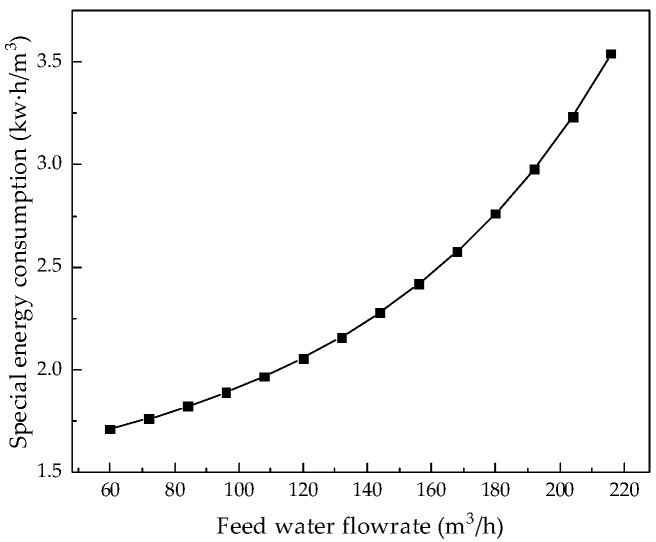
Effects of changes in feed water flowrate on the specific energy consumption.

During the operation of the coal-fired power plants, the feed water flowrate of the RO modules is unstable because the RO modules are not always in operation. It can be seen from Table 1 that the maximum flowrate of the membrane module is 240.0 m^3^/h, so, keeping other parameters unchanged, the influence of the membrane modules’ feed water flowrate, from 60.0 m^3^/h to 216.0 m^3^/h, on system performance can be analyzed. The analysis results for permeate flux, water production, water recovery rate, salt rejection rate, and specific energy consumption are shown in Figure 8, Figure 9, Figure 10 and Figure 11.

Analysis of the above results show that with the increase in the feed water flowrate of the RO modules, the permeate flux increases, which leads to an increase in water production, but if the feed water flowrate is too high, the water production becomes stable or even decreases. The reason for the above phenomenon is that the velocity in the feed channel is positively related to the feed water flowrate so that when the feed water flowrate increases, the flowrate on the membrane surface increases correspondingly. When the flowrate is less than 180 m^3^/h, the wastewater in the pipeline has enough time to penetrate, so water production increases with the increase in the flowrate. However, when the feed water flowrate is greater than 180 m^3^/h, the water does not have enough time to penetrate, resulting in a decrease in water production.

Figure 10 and Figure 11 analyze the effect of feed water flowrate on system performance parameters. Figure 11 shows that with an increase in the feed water flowrate, the water recovery rate of the system decreases, while the salt rejection rate increases and the growth rate of the salt rejection rate continues to slow down. Similarly, with the continuous increase in flowrate, the residence time of the feed wastewater in the pipeline is greatly shortened. In this case, neither water nor salt has enough time to pass to the permeate side, so the water recovery rate decreases. As the feed water flowrate increases, the velocity at the membrane surface increases, so the pressure rises, reducing the concentration polarization, and thereby the salt rejection rate is slightly increased. Figure 11 shows that as the feed water flowrate increases, the specific energy consumption also increases. With an increase in the feed water flowrate, the velocity at the membrane surface increases, the pressure in the pipeline rises, and more energy consumption is needed to produce water per unit volume.

#### 4.2.2. Effect of Feed Temperature on System Performance

**Figure 12:**
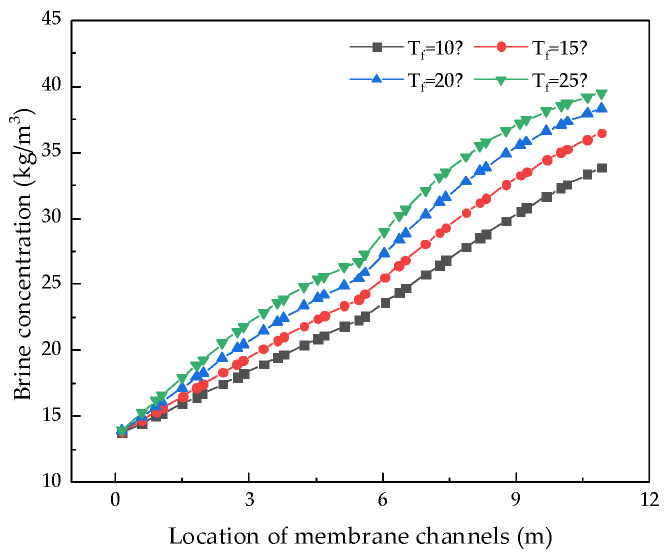
Effect of changes in feed water temperature on brine concentration.

**Figure 13:**
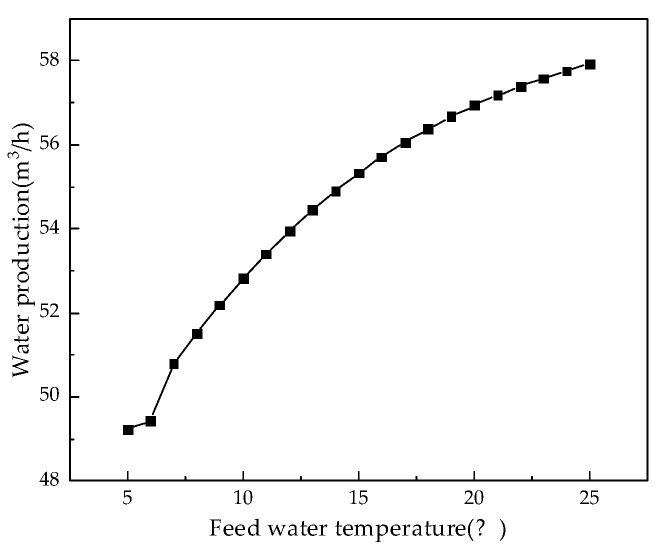
Effect of changes in feed water temperature on water production.

**Figure 14:**
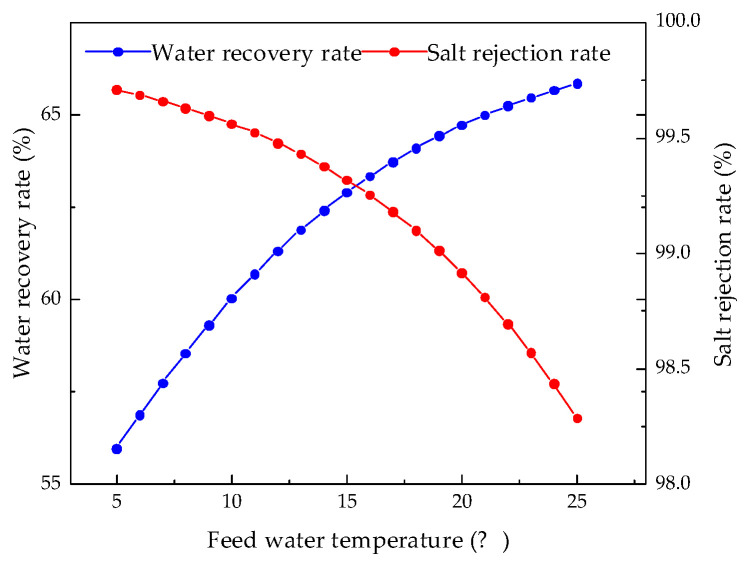
Effects of feed water temperature changes on water recovery rate and salt rejection rate.

**Figure 15:**
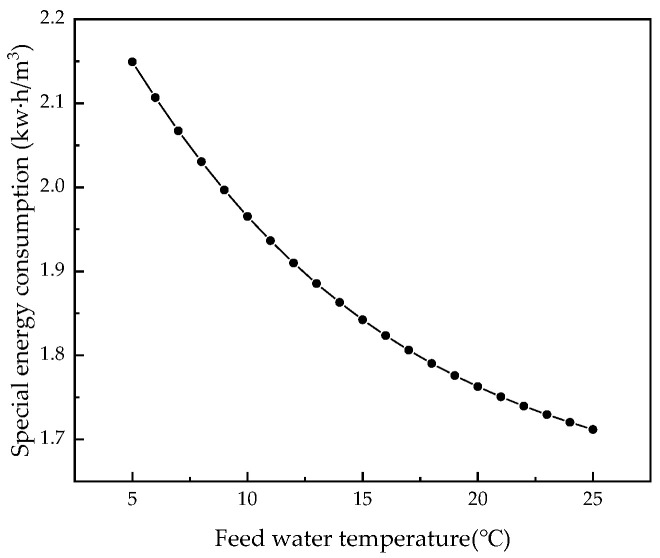
Effect of changes in feed water temperature on specific energy consumption.

The RO membrane is very sensitive to changes in the feed water temperature. Therefore, we kept other parameters unchanged and analyzed the influence of feed temperature from 5 °C to 25 °C on system performance. Figure 12, Figure 13, Figure 14 and Figure 15 illustrate the effects of feed water temperature on RO system performance.

It can be seen from Figure 12 and Figure 13 that as the feed water temperature increases, the brine concentration in the membrane channel increases accordingly. The water production of the RO modules also increases, and the water production increases (or decreases) by 0.3% to 2.5% for every 1 °C increase (or decrease) in the feed water temperature. Since, with an increase in the feed water temperature, the viscosity of water molecules decreases and the diffusivity increases, and with an increase in the moving speed, the water production increases, the brine concentration in the membrane channel also increases. Figure 14 and Figure 15 illustrate the effects of temperature on system performance. As the temperature increases, the water recovery rate of the system increases, while the salt rejection rate and specific energy consumption decrease. An increase in the feed water temperature also accelerates the movement of molecules so that the membrane modules pass through more water and salt, and as the water recovery rate of the system increases, so the concentration of brine in the pipeline will increase. An increase in permeated salt is accompanied by a decrease in the salt rejection rate. The higher the temperature, the faster the water molecules move and the less energy is required.

#### 4.2.3. Effect of Feed Water Pressure on System Performance

**Figure 16:**
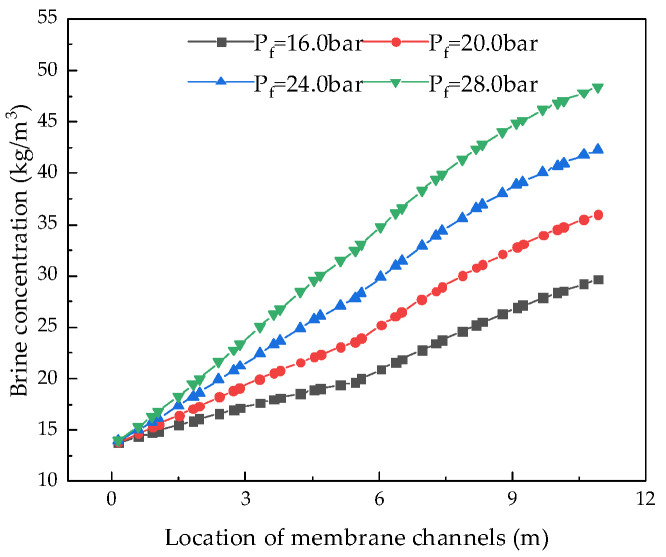
Effect of changes in feed pressure on brine concentration.

**Figure 17:**
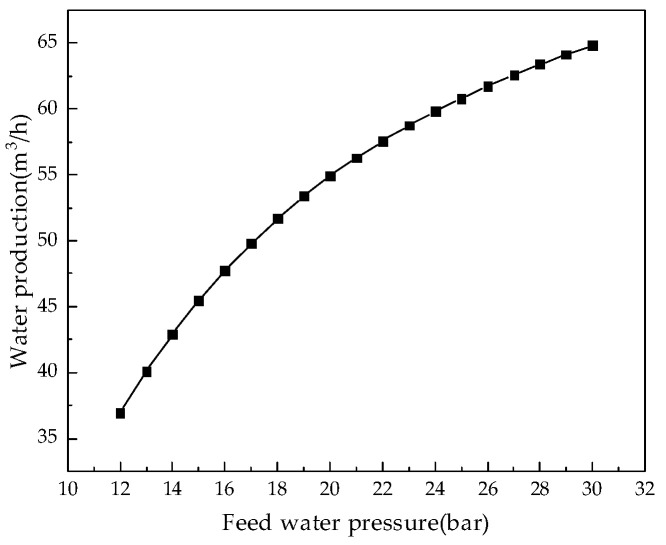
Effect of changes in feed water pressure on water production.

**Figure 18:**
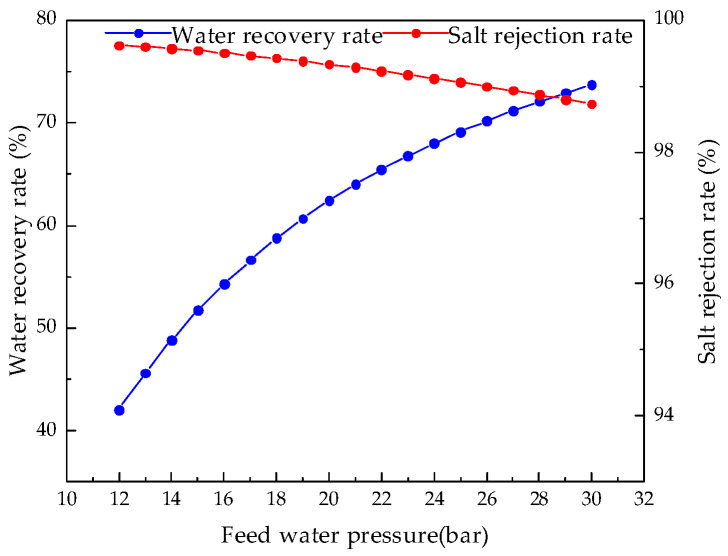
Effects of changes in feed water pressure on water recovery and salt rejection rate.

**Figure 19:**
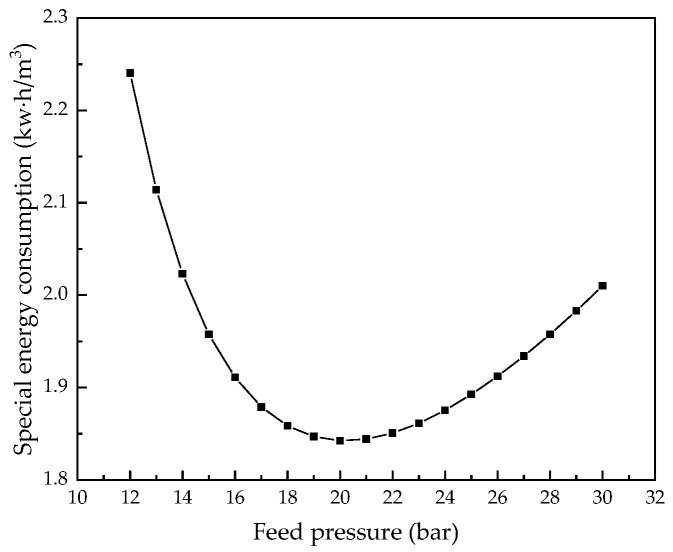
Effect of changes in feed water pressure on specific energy consumption.

Keeping other parameters unchanged, we analyzed the influence of feed water pressure from 12.0 bar to 30.0 bar on system performance. Figure 16, Figure 17, Figure 18 and Figure 19 illustrate the effects of feed water pressure on RO system performance.

From the analysis of the above figures, it can be seen that an increase in the feed water pressure increases the brine concentration in the membrane channel, water production, and the water recovery rate of the system. The increase in feed water pressure increases the driving force of the RO module, resulting in an increase in water production. The increased permeate water dilutes the salts permeating the membrane, resulting in a higher water recovery rate. With an increase in pressure, the excessively high salt rejection rate increases the concentration polarization, which leads to an increase in salt permeation, which offsets the increased water production, resulting in a slight decrease in the desalination rate. As the feed water pressure increases, the specific energy consumption first decreases and then increases, so there is a minimum value. As the net pressure driving the RO modules increases, the specific energy consumption required to generate a unit volume of permeate water decreases. When the feed water pressure exceeds a certain value, the concentration polarization is increased, which increases the specific energy consumption.

#### 4.2.4. Effect of Feed Concentration on System Performance

**Figure 20:**
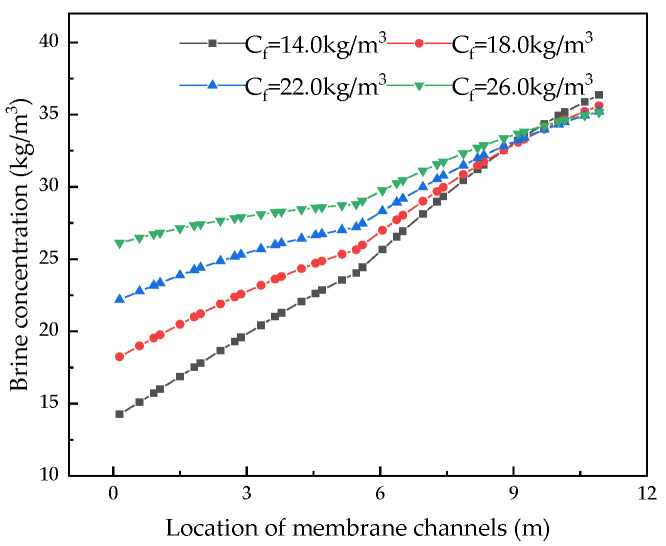
Effects of changes in feed concentration on brine concentration.

**Figure 21:**
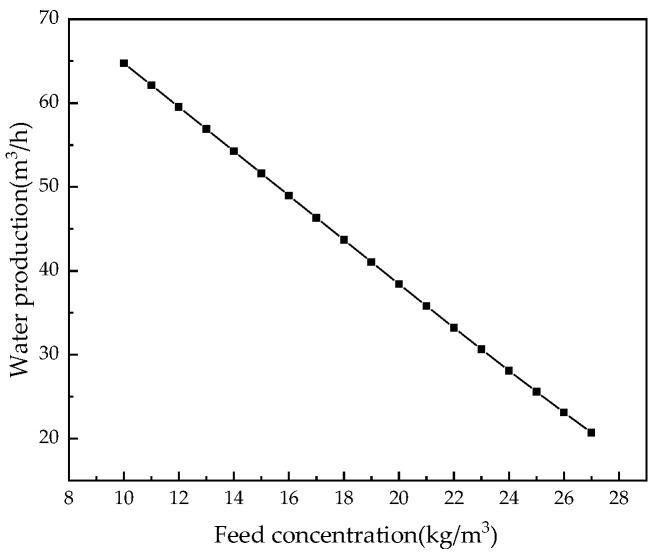
Effect of changes in feed concentration on water production.

**Figure 22:**
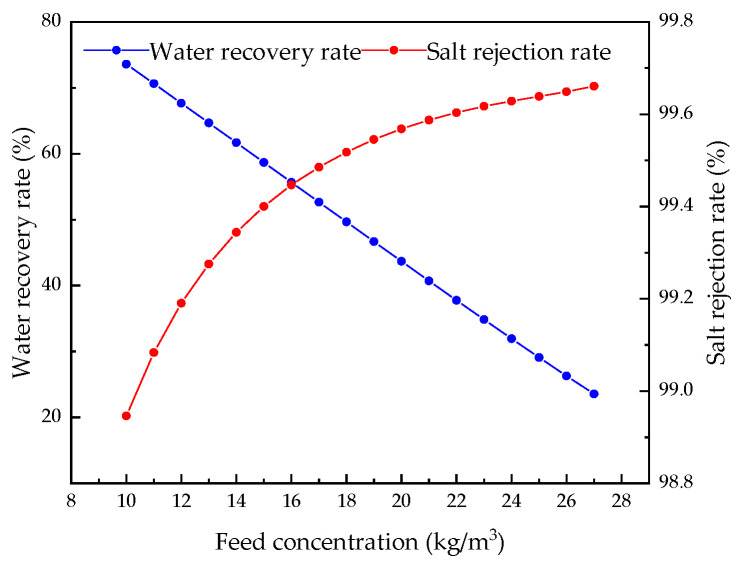
Effects of changes in feed concentration on water recovery and salt rejection rate.

**Figure 23:**
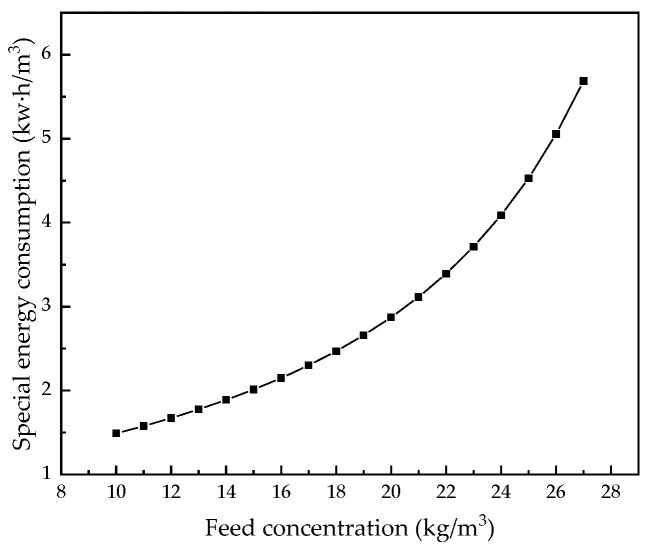
Effect of changes in feed concentration on specific energy consumption.

Keeping other parameters unchanged, we analyzed the influence of feed concentration from 10.0 kg/m^3^ to 27.0 kg/m^3^ on the system. Figure 20, Figure 21, Figure 22 and Figure 23 demonstrate the effect of feed pressure on RO system performance.

Figure 20 and Figure 21 show that as the feed concentration increases, the osmotic pressure increases, while the driving force remains unchanged, resulting in a linear downward trend in water production and an increase in brine concentration in the membrane channel, which eventually converges. This is because, with the increase in the feed concentration, the osmotic pressure of the feed water becomes higher and the driving force decreases, thereby reducing water production.

Figure 22 and Figure 23 show that, as the feed concentration increases, water production decreases, resulting in a decrease in water recovery rate. With an increase in feed concentration, a small amount of salt is permeated, but due to the tight structure of the RO membrane and the rapid decline in water production, the permeated salt is offset so that the salt rejection rate of the system increases slightly. The increase in the osmotic pressure of the water supply and the decrease in the driving force require more energy consumption per unit volume of water, so the specific energy consumption increases.

## 5. Operational Optimization of the Membrane Treatment Process for Coal-Fired Power Plant Wastewater

Through the above simulation analysis, it is clear that the goal of the lowest specific energy consumption and daily average operating cost of the system can be achieved by adjusting the feed conditions of the system. Considering the actual operation of the coal-fired power plant wastewater treatment system, the variables for optimal operation are mainly the controllable factors of the RO system (the feed water pressure, the feed water flowrate, and the pressure of the booster pump), but uncontrollable factors (feed water temperature and feed water concentration) can also affect the performance of the system.

Therefore, in the operational optimization analysis for coal-fired power plant wastewater treatment, the feed temperature was selected as 15 °C and 25 °C, respectively, and the feed concentration was selected as 13.0 kg/m^3^ and 20.0 kg/m^3^, respectively. The optimization analysis of the lowest specific energy consumption and the lowest average daily operating cost under different working conditions was then carried out.

### 5.1. Operational Optimization Based on the Lowest Specific Energy Consumption

#### 5.1.1. Establishment of the Optimization Proposition

The minimum specific energy consumption is the optimization goal, and its optimization proposition can be expressed as follows:

Objective function:

$$\underset{P_{f},Q_{f},P\_boost}{Min}SEC$$

Constraints:

RO process model:

f(x,u,v)=0

Device constraints:

$$\begin{array}{l} P_{f}^{L}\leq P_{f}\leq P_{f}^{U}\\ Q_{f}^{L}\leq Q_{f}\leq Q_{f}^{U}\\ P\_boost^{L}\leq P\_boost\leq P\_boost^{U} \end{array}$$

Superficial feed velocity for RO modules:

$$V_{f}^{L}\leq V_{f}\leq V_{f}{}^{U}$$

Initial conditions:

$$P_{b}=\left\{ \begin{array}{l} P_{f},z=0\\ P_{r},z=L \end{array}\right.$$

$$C_{b}=\left\{ \begin{array}{l} C_{f},z=0\\ C_{r},,z=L \end{array}\right.$$

The superscripts *L* and *U* refer to the upper and lower limits of the parameters, and the f(x,u,v)=0 equation represents the RO process model of the system. The upper and lower limits of the constraints are shown in Table 5.

#### 5.1.2. Running Optimization Analysis

Based on the operational optimization proposition with the lowest specific energy consumption, solvers such as IPOPT and CONOPT are called through the GAMS platform to optimize the solution with the lowest specific energy consumption as the optimization goal. Under the setting parameters of the RO system for coal-fired power plant wastewater treatment, the following three cases are considered, which are solved through the GAMS platform, and the changes in system performance before and after optimization are compared:

Case 1: The fixed feed water temperature is 15 °C and the feed concentration is 13.6 kg/m^3^.

Case 2: The fixed feed water temperature is 25 °C and the feed concentration is 13.6 kg/m^3^.

Case 3: The fixed feed water temperature is 15 °C and the feed concentration is 20.0 kg/m^3^.

A comparative analysis before and after optimization is carried out for the above three situations. Before optimization, the fixed feed water pressure of the RO system for coal-fired power plant wastewater is 20.3 bar, the feed water flowrate is 88.0 m^3^/h, and the pressure of the booster pump is 12.0 bar. After optimization, considering the safety and actual operation of the coal-fired power plant wastewater RO system, the upper and lower bounds of the operating pressure, the feed water flowrate, and the pressure of the booster pump are given in Table 5, and, based on the optimization proposition with the lowest specific energy consumption, the RO process is solved on the GAMS platform and the performance parameters of the system before and after optimization are compared.

Case 1 is optimized and solved; the optimal feeding conditions are obtained: the feed water pressure is 17.57 bar, the feed water flowrate is 72.1 m^3^/h, and the pressure of the booster pump is 14.12 bar. Table 6 presents the comparison results before and after Case 1 system optimization. Compared with the values before optimization, the water recovery rate of the optimized system increases by 11.2%, the salt rejection rate decreases by 0.8%, and the specific energy consumption decreases by 0.431 kw·h/m^3^, which is 23.6% lower than the original. The water recovery rate of the optimized system has been significantly improved, and the specific energy consumption has been significantly reduced, achieving the goal of reducing energy consumption. The salt rejection rate of the system has been reduced by 0.8%, which is within an acceptable range. However, the performance of the system is greatly improved.

The temperature in Case 2 is 25 °C and the rest of the parameters remain unchanged. Case 2 is optimized and solved, and the optimal feeding conditions are obtained as follows: the feed water pressure is 21.28 bar, the feed water flowrate is 91.0 m^3^/h, and the pressure of the booster pump is 12.41 bar. Table 7 shows the comparison results before and after system optimization. Before and after system optimization, the water recovery rate of the system increases by 9.0%, the salt rejection rate decreases by 1.7%, and the specific energy consumption decreases by 0.319 kw·h/m^3^, which is 18.6% lower than the original. Overall, the performance of the optimized system is also greatly improved.

The feed concentration in Case 3 is 20.0 kg/m^3^ and other parameters remain unchanged. Case 3 is optimized and solved, and the optimal feeding conditions are obtained as follows: the feed water pressure is 20.25 bar, the feed water flowrate is 57.1 m^3^/h, and the pressure of the booster pump is 11.52 bar. Table 8 shows the comparison results before and after system optimization. Before and after the system optimization, the water recovery rate of the system increases by 20.7%, the salt rejection rate decreases by 2.0%, and the specific energy consumption decreases by 1.19 kw·h/m^3^, which is 42.6% lower than the original. Although the salt rejection rate of the optimized system is reduced by 2.0%, the overall performance of the system is greatly improved.

Combining the above three optimized systems with different operating conditions, under the circumstance that the salt rejection rate of the optimized coal-fired power plant wastewater does not decrease by more than 2.0%, the water recovery rate and specific energy consumption of the system have been significantly improved. The purpose of reducing the energy consumption of the system has been achieved.

### 5.2. Operational Optimization Based on the Lowest Average Daily Operating Cost

#### 5.2.1. Economic Model of Coal-Fired Power Plant Wastewater Treatment Operation Cost

To achieve the goal of the lowest average daily operating cost, firstly, it is indispensable to establish a correct economic model of the operating cost of coal-fired power plant wastewater treatment. Secondly, it is necessary to establish the correct optimization proposition. The optimization solution is carried out through the GAMS platform and the optimal strategy for the operational optimization of coal-fired power plant wastewater treatment can be obtained. The operating cost of the coal-fired power plant wastewater membrane treatment system can be divided into the following main component costs:

1. The operating costs of the feed water intake and pretreatment system mainly include: ① chemical addition cost (*OC_CH_*), including acid, added scale inhibitors and flocculants, and other additives required to change hardness and composition; and ② water intake energy cost (*OC_IP_*).
2. Operating electricity (energy) consumption (*OC_EN_*), including the energy consumption of coal-fired power plant wastewater intake systems, high-pressure pumps, and booster pumps.
3. RO membrane replacement cost (*OC_ME_*). According to the design and operating conditions, it is calculated at a replacement rate of about 15–20%.
4. Maintenance cost (*OC_MN_*), including the maintenance costs for each element of the entire system, such as membrane, high pressure pump, booster pump, motor, etc.
5. Labor cost (*OC_LB_*).

Among the above operating costs, the cost of chemical additives, water intake energy consumption, and operating energy consumption are related to the flowrate of the system, while the membrane replacement cost, maintenance cost, and labor cost are independent of flowrate. The specific operating costs corresponding to each part are as follows [34]:

Chemical additive cost:

(33) $$OC_{CH}=kQ_{f}$$

where *k* is the conversion factor of chemical additive cost and *Q_f_* represents the feed water flowrate of the RO system.

Water consumption cost:

(34) $$OC_{IP}=\frac{P_{o}\cdot Q_{f}\cdot P_{elc}}{\eta_{IP}}\times PLF$$

Here, *P_0_* refers to the outlet pressure of the intake pump, *Q_f_* represents the feed water flowrate of the RO system, *PLF* is the load factor, *P_elc_* denotes the electricity price, and $\eta_{Ip}$ is the efficiency of the coal-fired power plant wastewater intake pump.

Operating energy cost:

(35) $$OC_{EN}=\left[\frac{P_{f}Q_{f}/(\varepsilon_{p}\cdot \varepsilon_{VFD})+P\_boost\cdot Q_{f2}/\varepsilon_{bp}}{Q_{p}}\right]\cdot P_{elc}$$

The operating energy cost mainly includes the energy consumption of the high-pressure pump and the booster pump. *P_f_* represents the feed water pressure of the RO system. *Q_f2_* and *Q_p_* are the feed water flowrate of the second-stage RO module and the final water flowrate of the RO system, respectively.

RO membrane replacement cost:

(36) $$OC_{ME}=Pri_{ME}\cdot NM\cdot \zeta_{re}/360$$

where $Pri_{ME}$ denotes the unit price of RO membrane elements, *NM* is the total number of membrane elements of the RO system, and $\zeta_{re}$ represents the replacement rate of membrane modules.

When studying the average daily operating cost of the RO system for coal-fired power plant wastewater treatment, the RO cleaning and maintenance costs can be ignored, so the maintenance cost of the system can be expressed as the following equation:

Maintenance cost:

(37) $$OC_{MN}=OC_{MNCON}=\omega OC_{RO}$$

Labor cost:

(38) $$OC_{LB}=Pri_{LB}\cdot N_{LB}$$

(39) $$N_{LB}=Q_{p}\cdot NP/100$$

ω is the proportion of maintenance costs, *NLB* refers to the number of labor tasks, and *Qp* represents the final water flowrate of the RO system.

Total operating cost:

(40) $$\begin{array}{l} OC=OC_{IP}+OC_{EN}+OC_{MER}+OC_{MN}+OC_{CH}+OC_{LB}\\ \;=OC_{IP}+OC_{EN}+OC_{MER}+OC_{MNCON}+OC_{CH}+OC_{LB}\\ \;=OC_{IP}+OC_{EN}+OC_{MER}+0.03OC_{RO}+OC_{CH}+OC_{LB}\\ \;=\left(OC_{IP}+OC_{EN}+OC_{MER}+OC_{CH}+OC_{LB}\right)/0.97 \end{array}$$

#### 5.2.2. Establishment of the Optimization Proposition

The lowest average daily operating cost of the coal-fired power plant wastewater RO system is the optimization goal, and its optimization proposition can be expressed as follows:

Objective function:

$$\underset{P_{f},Q_{f},P\_boost}{Min}OC$$

Constraints:

RO process model:

f(x,u,v)=0

Operational cost model:

g(x,u,v)=0

Device constraints:

$$\begin{array}{l} P_{f}^{L}\leq P_{f}\leq P_{f}^{U}\\ Q_{f}^{L}\leq Q_{f}\leq Q_{f}^{U}\\ P\_boost^{L}\leq P\_boost\leq P\_boost^{U} \end{array}$$

Superficial feed velocity for RO modules:

$$V_{f}^{L}\leq V_{f}\leq V_{f}{}^{U}$$

Initial conditions:

$$P_{b}=\left\{ \begin{array}{l} P_{f},z=0\\ P_{r},z=L \end{array}\right.$$

$$C_{b}=\left\{ \begin{array}{l} C_{f},z=0\\ C_{r},,z=L \end{array}\right.$$

The superscripts *L* and *U* refer to the upper and lower limits of the parameters, the equation f(x,u,v)=0 represents the RO process model of the system, and g(x,u,v)=0 represents the operational cost model of the system.

#### 5.2.3. Running Optimization Analysis

Based on the operational optimization proposition with the lowest average daily operating cost, solvers such as IPOPT and CONOPT are called through the GAMS platform to optimize the solution with the lowest average daily operating cost as the optimization goal. Under the setting parameters of the RO system for the coal-fired power plant wastewater treatment, the three operating conditions with the same optimization proposition with the lowest specific energy consumption are solved by the GAMS platform and the changes in the average daily operating costs of the system before and after optimization are compared:

Case 1: The fixed feed water temperature is 15 °C and the feed concentration is 13.6 kg/m^3^.

Case 2: The fixed feed water temperature is 25 °C and the feed concentration is 13.6 kg/m^3^.

Case 3: The fixed feed water temperature is 15 °C and the feed concentration is 20.0 kg/m^3^.

Before optimization, the fixed feed water pressure of the RO system for coal-fired power plant wastewater treatment is 20.3 bar, the feed water flowrate is 88.0 m^3^/h, and the pressure of the booster pump is 12.0 bar. After optimization, the upper and lower bounds of the feed water pressure, feed water flowrate, and booster pump pressure are given based on the consideration of the safety and actual operation of the coal-fired power plant wastewater RO system. Based on the optimization proposition with the lowest average daily operating cost, the RO process is solved on the GAMS platform and the performance parameters of the system before and after optimization are compared.

Among the system operating costs, labor costs, system maintenance costs, and membrane replacement costs have nothing to do with flowrate, so these values are fixed before and after optimization, but the proportions of these values change due to changes in other costs. Correspondingly, the energy consumption cost of pretreatment, chemical cost, and operating energy cost are related to the feed parameters of the system, so these values vary before and after optimization.

**Figure 24:**
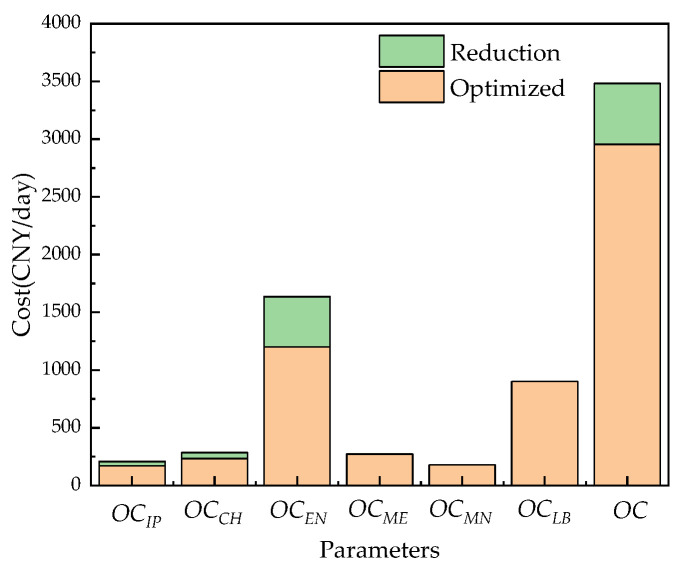
Comparison of the average daily operating costs of the system before and after optimization.

Table 9 and Figure 24 show the comparison results before and after system optimization. Under the operation of Case 1, the overall operating cost of the system before optimization is 3481.8 CNY/day, the cost of chemical agents accounts for 8.2%, the cost of operating energy accounts for 47.0%, and the cost of pretreatment energy accounts for 6.0%. After optimization, the overall operating cost of the system is 2954.9 CNY/day, which is 15.2% lower than that before optimization. The cost of chemical agents is reduced by 18.4%, the cost of operating energy consumption is reduced by 26.7%, and the cost of RO pretreatment energy consumption is reduced by 18.4%. It can be clearly seen that the operating cost of the RO system has been greatly reduced after the optimization of the RO system. The orange shading in Figure 24 represents the daily average operation cost of the RO system after optimization, the green represents the reduction in the daily average cost after optimization, and the total height represents the daily average operating cost before system optimization.

**Figure 25:**
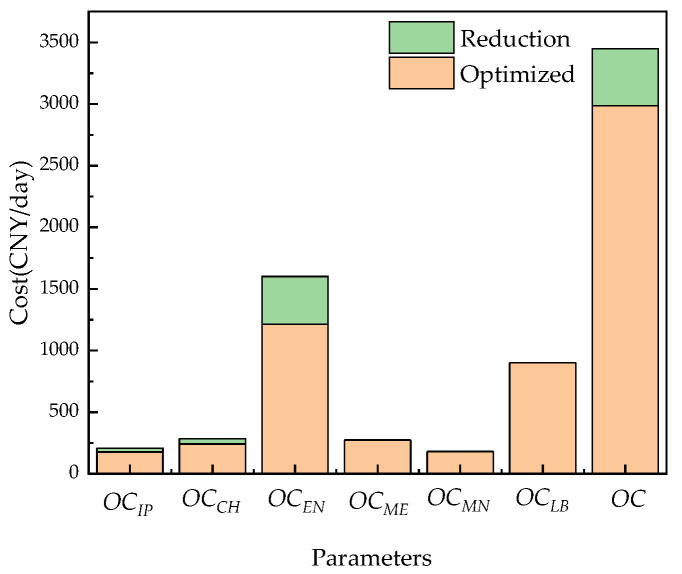
Comparison of the average daily operating costs of the system before and after optimization.

Table 10 and Figure 25 show the results before and after system optimization. Under the operation of Case 2, the overall operating cost of the system before optimization is 3447.3 CNY/day, the cost of chemical agents, the energy cost of the RO process, and the cost of operating energy consumption accounts for 8.3%, 52.4%, and 46.4%, respectively. After optimization, the total system operating cost is 2984.9 CNY/day, which is 13.4% lower than that before optimization. The cost of chemical agents is reduced by 15.0%, the operating energy consumption cost reduced by 15.0%, and there is an 18.4% reduction in pretreatment energy costs. It can be clearly seen that the operating cost of the RO system has been greatly reduced after the optimization of the RO system.

**Figure 26:**
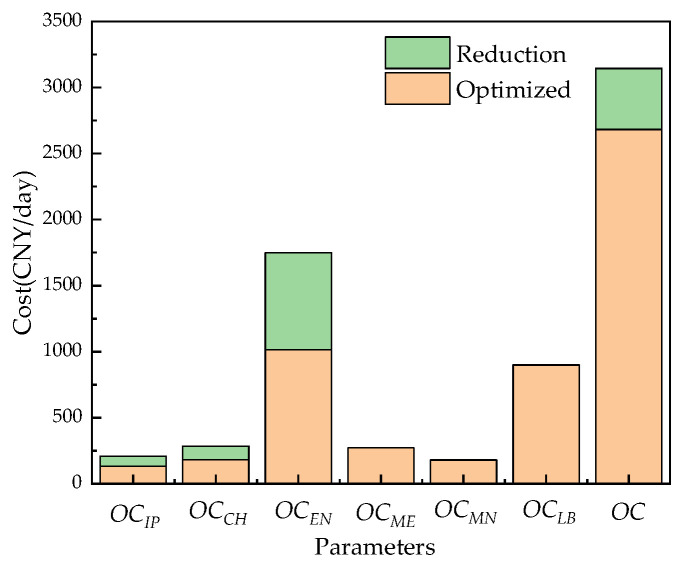
Comparison of the average daily operating costs of the system before and after optimization.

Table 11 and Figure 26 show the results before and after system optimization. Under the operation of Case 3, the overall operating cost of the system before optimization is 3593.7 CNY/day, the cost of chemical agents accounts for 7.9%, the cost of operating energy accounts for 48.6%, the cost of pretreatment energy consumption accounts for 5.8%. After optimization, the overall operating cost of the system is 2682.6 CNY/day, which is 25.4% lower than that before optimization. The cost of chemical agents is reduced by 36.3%, the cost of operating energy consumption is reduced by 41.9%, and the cost of RO pretreatment energy consumption is reduced by 36.3%. It can be clearly seen that the operating cost of the RO system has been greatly reduced after the optimization of the RO system.

In summary, the increase in feed temperature reduces the average daily operating energy consumption of the system and the increase in feed concentration increases the daily average operating energy consumption of the system. For the operational optimization analysis under the above three different operating conditions, after the optimization, the daily average total operating cost of the system has decreased significantly, reaching the goal of reducing the average daily operating cost.

## 6. Conclusions

RO technology is important not only for seawater desalination but also for coal-fired power plant wastewater treatment. In this paper, a coal-fired power plant system in Inner Mongolia was taken as an example to study the simulation and optimization of the membrane treatment process of coal-fired power plant wastewater. Firstly, based on the solution–diffusion theory, pressure drop, and osmotic concentration polarization phenomena, a mechanistic model equation of the RO process of coal-fired power plant wastewater treatment was completed by drawing on the seawater desalination process. Through the correction of model parameters, a system model suitable for coal-fired power plant wastewater treatment was obtained. Secondly, in view of the opaqueness of the resource treatment process, a simulation analysis of the coal-fired power plant wastewater treatment process was carried out and internal data for the coal-fired power plant wastewater RO treatment process were obtained, realizing the transparent management of the system. Finally, an optimization analysis of the coal-fired power plant wastewater treatment system was carried out.

Focusing on the problem of high energy consumption in the resource treatment of coal-fired power plant wastewater by the RO process, an optimization and analysis of a coal-fired power plant wastewater RO treatment system was carried out. To begin with, an optimization analysis of three different working conditions was performed, with the lowest specific energy consumption as the optimization goal. The water recovery rate was increased by 11.2%, 9.0%, and 20.7%, and the specific energy consumption was decreased by 23.6%, 18.6%, and 42.6%, respectively, which fully proved the effectiveness of the optimization strategy. Afterwards, taking the lowest average daily operating cost as the optimization goal, the same three working conditions were analyzed. The average daily operating costs of the system were reduced by 526.9 CNY/day, 462.4 CNY/day, and 911.1 CNY/day, which also proved the effectiveness of the optimization strategy. Therefore, the simulation and optimization research on the recycling process of coal-fired power plant wastewater presented here can promote the treatment of coal-fired power plant wastewater and is significant for the development of zero-emission coal-fired power plant wastewater treatment systems.

## Figures and Tables

**Table 1:** Membrane element property.

Membrane Property	Unit	Value
RO membrane model	——	PROC10
Membrane material	——	Aromatic polyamide composites
Maximum flowrate	m^3^/h	240
Maximum pressure	bar	41.4
Maximum temperature	°C	45
Maximum SDI	—	5
Effective area	m^2^	479.7
Effective membrane leaf length	m	0.91
Effective membrane leaf width	m	0.704
Inlet mesh thickness	mm	0.8636
Pump efficiency	——	0.75

**Table 2:** Water quality parameters of coal-fired power plant wastewater.

Property	Result (mg/L)	Property	Result
K^+^	89.00	Total alkalinity	16.4 mmol/L
Na^+^	4030.00	Alkalinity, PPM CaCO_3_	16.2 mmol/L
1/2Ca^2+^	268.54	Total silica	26.3 mg/L
1/2Mg^2+^	36.45	Inactive silicon	7.1 mg/L
NH_4_^+^	8.12	TDS	13,668.0 mg/L
1/2Sr^2+^	8.80	SS	8.0 mg/L
1/2Ba^2+^	0.11	Ammonia nitrogen	6.30 mg/L
1/3Fe^3+^	0.29	TUB	0.396 NTU
Cl^−^	2480.00	CODCr	51.0 mg/L
1/2SO_4_^2−^	5201.00	PH (25 °C)	8.27
HCO_3_^−^	964.12	Water temperature	15 °C
1/2CO_3_^2−^	12.00	Water pressure	20.8 bar
NO_3_^−^	445.9	Water flowrate	88.0 m^3^/h
F^−^	6.90	Water concentration	13,600.0 mg/L

**Table 3:** Data comparison.

Data Sources	Concentration of the First-Stage Permeate (m^3^/h)	Concentration of the Second-Stage Permeate (m^3^/h)	Final Product Water Concentration (m^3^/h)	Second-Stage Inlet Pressure (bar)
Model data before correction	0.155	0.140	0.148	31.0
Corrected model data	0.228	0.489	0.308	32.1
IMSDesign’s data	0.192	0.495	0.289	31.4

**Table 4:** Comparison of power plant data and simulation data.

Properties	Data 1		Data 2		Data 3	
	Power Plant Data	GAMS Data	Power Plant Data	GAMS Data	Power Plant Data	GAMS Data
Feed water flowrate of RO modules (m^3^/h)	64.4383	64.4383	72.7411	72.7411	79.7619	79.7619
Feed water pressure of the first stage (bar)	22.75	22.75	22.7885	22.7885	21.7115	21.7115
Feed pump outlet conductivity ${EC}_{25\text{°}C}\;(\mu S/\mathrm{cm})$	19,887.1	19,887.1	17,994.5	17,994.5	16,712.5	16,712.5
Water production flowrate of the first stage (m^3^/h)	—	34.9644	—	41.4348	—	45.0576
Water production flowrate of the second stage (m^3^/h)	—	7.1106	—	8.1844	—	9.8966
RO module water production flowrate (m^3^/h)	44.8824	42.0750	52.6563	49.6192	55.6918	54.9542
Feed water flowrate of RO modules booster pump (m^3^/h)	27.3962	29.4744	30.5708	31.3068	41.5293	34.7040
Concentrate water flowrate of RO modules (m^3^/h)	19.7955	22.3636	21.9017	23.1224	25.4426	24.8073
Water production pressure in the first stage (bar)	22.2885	22.2993	23.2885	22.2517	21.3269	21.0693
Concentrated water pressure of RO modules (bar)	28.4423	29.0406	29.7500	28.9632	28.4423	29.0406
Salt rejection rate (%)	97.4835	98.6559	98.7274	98.7235	97.4835	98.6559

**Table 5:** Operation limits.

Property	Value
Maximum feed water pressure (bar)	41.4
Maximum feed water flowrate (m^3^/h)	240
Minimum feed water flowrate (m^3^/h)	24
Maximum pressure of booster pump (bar)	30
∅	1.5
Maximum superficial feed velocity (m/s)	0.38
Minimum superficial feed velocity (m/s)	0.038

**Table 6:** Performance comparison results before and after system optimization in Case 1.

System Performance Parameters	Before Optimization	Optimized
Water recovery rate (%)	63.3%	74.5%
Salt rejection rate (%)	99.3%	98.5%
Specific energy consumption (kw·h/m^3^)	1.826	1.395

**Table 7:** Performance comparison results of the Case 2 system before and after optimization.

System Performance Parameters	Before Optimization	Optimized
Water recovery rate (%)	66.0%	75.0%
Salt rejection rate (%)	98.2%	96.5%
Specific energy consumption (kw·h/m^3^)	1.715	1.396

**Table 8:** Performance comparison results of Case 3 system before and after optimization.

System Performance Parameters	Before Optimization	Optimized
Water recovery rate (%)	44.2%	64.9%
Salt rejection rate (%)	99.6%	97.6%
Specific energy consumption (kw·h/m^3^)	2.795	1.605

**Table 9:** Operational composition before and after RO system optimization.

Related Parameters	Before Optimization		After Optimization	
	Value (CNY/Day)	Proportion (%)	Value (CNY/Day)	Proportion (%)
Water intake energy cost (*OC_IP_*)	208.0	6.0	169.8	5.7
Chemical cost (*OC_CH_*)	285.0	8.2	232.6	7.9
Running energy cost (*OC_EN_*)	1635.7	47.0	1199.4	40.6
RO membrane replacement cost (*OC_ME_*)	273.1	7.9	273.1	9.2
Maintenance cost (*OC_MN_*)	180.0	5.1	180.0	6.1
Labor costs (*OC_LB_*)	900.0	25.8	900.0	30.5
Total cost of RO system (*OC*)	3481.8	100.0	2954.9	100.0

**Table 10:** Operational composition before and after RO system optimization.

Related Parameters	Before Optimization		After Optimization	
	Value (CNY/Day)	Proportion (%)	Value (CNY/Day)	Proportion (%)
Water intake energy cost (*OC_IP_*)	208.0	6.0	176.7	5.9
Chemical cost (*OC_CH_*)	285.0	8.3	242.2	8.1
Running energy cost (*OC_EN_*)	1601.2	46.4	1212.9	40.6
RO membrane replacement cost (*OC_ME_*)	273.1	8.0	273.1	9.2
Maintenance costs (*OC_MN_*)	180.0	5.2	180.0	6.0
Labor costs (*OC_LB_*)	900.0	26.1	900.0	30.2
Total cost of RO system (*OC*)	3447.3	100.0	2984.9	100.0

**Table 11:** Operational composition before and after RO system optimization.

Related Parameters	Before Optimization		After Optimization	
	Value (CNY/Day)	Proportion (%)	Value (CNY/Day)	Proportion (%)
Water intake energy cost (*OC_IP_*)	208.0	5.8	132.6	4.9
Chemical cost (*OC_CH_*)	285.0	7.9	181.6	6.8
Running energy cost (*OC_EN_*)	1747.6	48.6	1015.3	37.9
RO membrane replacement cost (*OC_ME_*)	273.1	7.6	273.1	10.2
Maintenance costs (*OC_MN_*)	180.0	5.0	180.0	6.7
Labor costs (*OC_LB_*)	900.0	25.1	900.0	33.5
Total cost of RO system (*OC*)	3593.7	100.0	2682.6	100.0

## Data Availability

Data is contained within the article.
